# Fragment Exchange Plasmid Tools for CRISPR/Cas9-Mediated Gene Integration and Protease Production in Bacillus subtilis

**DOI:** 10.1128/AEM.02090-20

**Published:** 2020-12-17

**Authors:** Antonio García-Moyano, Øivind Larsen, Sushil Gaykawad, Eleni Christakou, Catherine Boccadoro, Pål Puntervoll, Gro Elin Kjæreng Bjerga

**Affiliations:** a NORCE Norwegian Research Centre, Bergen, Norway; University of Buenos Aires

**Keywords:** *Bacillus subtilis*, CRISPR, FX cloning, genome editing, subtilisin, protease, microbial fermentation

## Abstract

Since its discovery as part of the bacterial adaptative immune system, CRISPR/Cas has emerged as the most promising tool for targeted genome editing over the past few years. Various tools for genome editing in Bacillus subtilis have recently been developed, expanding and simplifying its potential development as an industrial species. A collection of vectors compatible with high-throughput (HTP) fragment exchange (FX) cloning for heterologous expression in Escherichia coli and *Bacillus* was previously developed. This vector catalogue was through this work supplemented with editing plasmids for genome engineering in *Bacillus* by adapting two CRISPR/Cas plasmids to the cloning technology. The customized tools allow versatile editing at any chosen genomic position (single-plasmid strategy) or at a fixed genomic locus (double-plasmid strategy). The single-plasmid strategy was validated by deleting the *spoIIAC* gene, which has an essential role in sporulation. Using the double-plasmid strategy, we demonstrate the quick transition from plasmid-based subtilisin expression to the stable integration of the gene into the *amyE* locus of a seven-protease-deficient KO7 strain. The newly engineered B. subtilis strain allowed the successful production of a functional enzyme. The customized tools provide improvements to the cloning procedure, should be useful for versatile genomic engineering, and contribute to a cloning platform for a quick transition from HTP enzyme expression to production through the fermentation of industrially relevant B. subtilis and related strains.

**IMPORTANCE** We complemented a cloning platform with new editing plasmids that allow a quick transition from high-throughput cloning and the expression of new enzymes to the stable integration of genes for the production of enzymes through B. subtilis fermentation. We present two systems for the effective assembly cloning of any genome-editing cassette that shortens the engineering procedure to obtain the final editing constructs. The utility of the customized tools is demonstrated by disrupting *Bacillus*’ capacity to sporulate and by introducing the stable expression of subtilisin. The tools should be useful to engineer B. subtilis strains by a variety of recombination events to ultimately improve the application range of this industry-relevant host.

## Abstract (precis)

We complemented a cloning platform with new editing plasmids that allow a quick transition from high-throughput cloning and the expression of new enzymes to the stable integration of genes for the production of enzymes through B. subtilis fermentation. We present two systems for the effective assembly cloning of any genome-editing cassette that shortens the engineering procedure to obtain the final editing constructs. The utility of the customized tools is demonstrated by disrupting *Bacillus*’ capacity to sporulate and by introducing the stable expression of subtilisin. The tools should be useful to engineer B. subtilis strains by a variety of recombination events to ultimately improve the application range of this industry-relevant host.

## INTRODUCTION

Bacillus subtilis and its relatives have been widely used as bacterial hosts for the recombinant expression of heterologous proteins (1–3). Industrial fermentation of *Bacillus* has been attractive due to its lack of pathogenicity and toxic by-products and is thus regarded as a safe bacterium for food and feed applications (4). Moreover, its low nutritional demands, ability to produce a wide range of industrial enzymes, and excellent secretory capability, which minimizes the need for downstream processing (3, 4), are features that make it an excellent production host organism in industrial processes.

The ability of *Bacillus* to produce high yields of recombinant proteins was previously hampered by the degradation of the products by the host’s native proteases during vegetative growth and stationary phase (5, 6). Strains in which multiple protease genes had been deleted and/or inactivated have proven to be superior to wild-type strains for the production of foreign proteins (1). For example, B. subtilis KO7 (*Bacillus* Genetic Stock Center [BGSC]), a derivative of the genome-sequenced common laboratory strain PY79 (7, 8), was generated by sequentially eliminating the coding sequences of seven proteases using native competence, recombination with a knockout selection cassette, and successive Cre-driven removal of the resistance gene (D. R. Zeigler, unpublished data). All seven knockouts in KO7 are confirmed by Sanger sequencing. The strain KO7, however, still carries the gene *wprA*, which encodes a quality control exopeptidase that is constitutively expressed during vegetative growth (9) and known to be functional at the wall-membrane interface or in the wall itself, rather than being secreted into the culture medium (10). This seven-protease-deletion strain has several beneficial properties for industrial applications, including the absence of antibiotic resistance genes and integrated or extrachromosomal plasmids. It is also prototrophic, which means that it is able to grow efficiently in standard media. Strain KO7 is a good candidate production organism due to its clean genetic background (11).

Recombinant DNA technology involves the use of bacterial plasmids as shuttle vectors to carry and transfer genes for heterologous expression. Antibiotic resistance genes are commonly used as selection markers to prevent plasmid instability and the unnecessary growth of plasmid-free cells. The use of antibiotics, however, is not suitable in industrial processes, particularly for the production of food-grade enzymes, due to the contamination of equipment, biomass, and product. The current trend in industrial processes is to bypass the use of antibiotics by developing marker-free production systems. Integration of the target gene into the chromosome is therefore an attractive strategy since it ensures high stability without the use of any antibiotic selective pressure. Among several DNA-editing tools already available for genome engineering in B. subtilis (12–14), CRISPR/Cas9-based systems are known to be more efficient since the host does not have an activated nonhomologous end-joining (NHEJ) system during growth (15). Its survival upon the introduction of a double-strand break (DSB) by the Cas9 nuclease relies entirely on successful recombination with a repair template. This strong selection thus minimizes the screening or selection efforts (15). Recently described CRISPR/Cas9 systems rely on a single broad-host-range plasmid that assembles the Cas9 protein from Streptococcus pyogenes, a single guide RNA (sgRNA), the donor repair DNA template, and other elements in a single vector backbone (16–18). CRISPR/Cas9 systems have also shown versatility in different engineering strategies, such as small insertions and deletions (indels) but also large genome deletions or gene insertions (15, 17, 19). However, obtaining the final editing vectors is often elaborate and time-consuming due to multiple cloning steps (16, 19–21). The repair template and sgRNA must be designed for each experiment depending on the targeted locus. In addition, the insertion of fragments of substantial sizes, such as an entire gene, makes the integration plasmids large, thus affecting the transformation efficiency (22). Here, rapid and efficient molecular cloning tools are critical in simplifying genetic engineering procedures, and efforts are sought in order to shorten the engineering of the final editing vector (19, 21).

Advancements in methods for the high-throughput (HTP) generation of genetic constructs have flourished over the last decades (23). Common methods for the integration of gene fragments into or between plasmids are based on recombination and/or ligation-independent methods (24–26) (e.g., TOPO TA, Gateway cloning, Gibson assembly, and In-Fusion cloning) and restriction/ligation cloning based on nonpalindromic type IIS restriction sequences (27) (e.g., Golden Gate assembly). Fragment exchange (FX) cloning combines the use of type IIS restriction enzymes and counterselection based on CcdB toxicity against gyrase originally introduced by Gateway cloning (28). The approach is highly effective due to the directional cloning caused by the orientation of the restriction site (SapI) and its capacity to generate trinucleotide hangs outside its recognition site and the presence of a counterselection gene, *ccdB*. It thus facilitates the efficient directional insertion of the fragment of interest in a one-pot reaction while leaving minimal cloning seams of only a single extra amino acid on either side of the protein and promoting one-step selection for positive transformants (29). Previously, we have customized vectors compatible with the FX cloning technology for HTP cloning and the heterologous expression of enzymes in Escherichia coli (30) and B. subtilis (31).

The aim of this work was to expand the FX-compatible vector catalogue with efficient and versatile plasmids for CRISPR/Cas9-mediated gene editing in B. subtilis. A single-plasmid system was adapted to FX cloning for the editing of any locus of choice after a single cloning step. Such a system will enable metabolic engineering of B. subtilis by deletions or insertions. A double-plasmid system was customized for FX cloning to facilitate the efficient insertion of protein-encoding genes into a fixed genomic position within the *amyE* locus. Combined with the previously developed catalogue of FX-compatible vectors, this system encompasses a cloning platform for fast transitions from high-throughput protein expression to production by fermentation of B. subtilis. The tools will facilitate the cloning procedure prior to genomic engineering, ultimately speeding up rational engineering of industrially relevant B. subtilis and related strains.

## RESULTS

### Customization of a CRISPR/Cas9 vector to FX cloning for versatile and simplified engineering of B. subtilis.

**FIG 1:**
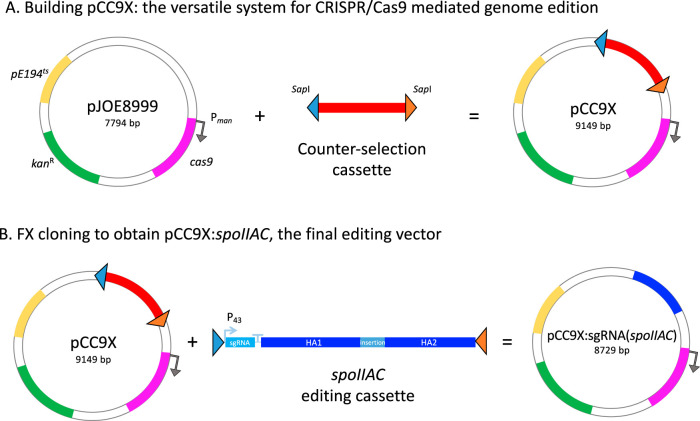
Customization of FX-compatible vectors for genome editing in B. subtilis. (A) The pCC9X plasmid was constructed by introducing an FX counterselection cassette into the backbone of pJOE8999, which contains a pUC minimal origin of replication for E. coli, the temperature-sensitive replication origin pET194^ts^ for B. subtilis, and a kanamycin resistance gene working in both organisms. The cassette harbors a lethal *ccdB* gene (indicated in red) flanked by two opposite outward-oriented SapI sites (triangles) useful for directional cloning. (B) An FX-compatible editing cassette is readily cloned into pCC9X by SapI restriction and ligation to obtain the final stable SapI-free editing vector, pCC9X:sgRNA(*spoIIAC*). The editing cassette is flanked by two opposite inward-oriented and compatible SapI sites (triangles) and contains an inducible sgRNA and a short insert between two homology arms (HA1 and HA2) against *spoIIAC*. Gene lengths are not to scale.

Using the shuttle vector pJOE8999 as a starting point, we customized it to FX cloning (28) by introducing a counterselection fragment featuring the lethal *ccdB* gene flanked by two SapI sites in opposite directions, according to the FX design (28), generating pCC9X (Fig. 1A). The vector design facilitates the directional insertion of any editing cassette containing FX-compatible SapI overhangs. For a CRISPR/Cas-based engineering approach, the editing cassette should contain an inducible sgRNA, and an insert sequence, flanked by two homology arms against the B. subtilis chromosome (17). The editing may cause frameshift mutations or sequence replacements. The editing cassette may constitute a linear DNA fragment or be inserted into the pINITIAL vector or equivalent entry vectors prior to subcloning (28, 29). Correctly cloned plasmids can be identified by an efficient selection process based on the elimination of *ccdB*. The final plasmid construct harbors the essential elements from the shuttle vector pJOE8999 (17), namely, a pUC minimal origin of replication for E. coli, the temperature-sensitive replication origin of pE194^ts^ for B. subtilis, and a kanamycin resistance gene working in both organisms. It also carries the *cas9* gene from S. pyogenes under the control of the mannose-inducible promoter (P*_man_*). Like pJOE8999, the customized vector should allow genome engineering of the surrogate host B. subtilis (Fig. 1A) albeit with an easier cloning procedure prior to the editing event.

### Generation of an asporogenic strain demonstrates the functionality of the FX-compatible single-plasmid system.

**FIG 2:**
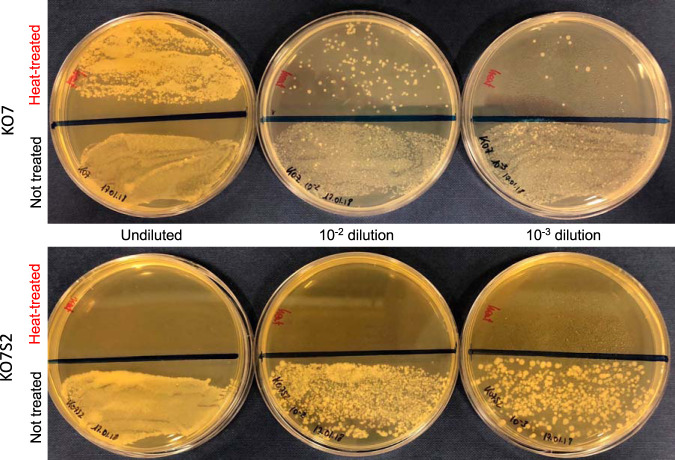
Demonstration of the asporogenic phenotype in B. subtilis KO7S2 introduced by using the FX-compatible pCC9X vector. The CRISPR/Cas9-based pCC9X:sgRNA(*spoIIAC*) plasmid was used to knock out the *spoIIAC* sporulation gene. Sporulation was induced by metabolic stress, and vegetative cells were killed by heat treatment. Only the wild-type KO7 strain produces colonies from heat-resistant spores in all serial dilutions (top halves of plates in the top panel), whereas KO7S2 did not (top halves of plates in the bottom panel). Nontreated cells (bottom halves of plates in both panels) served as controls of cell viability.

The validation of the single-plasmid system and its suitability for CRISPR/Cas9-driven gene engineering were originally shown by Altenbuchner (17). We demonstrate the feasibility of the FX-compatible version by knocking out a sporulation gene to generate an asporogenic *Bacillus* strain. The abruption of *Bacillus*’ ability to sporulate minimizes the risk of cross-contamination in microbial fermentation in a laboratory or industrial setting. We chose B. subtilis KO7 as a model strain for engineering due to its low protease background following the deletion of seven protease-encoding genes that minimize the risk of downstream proteolytic degradation of expressed proteins (Table 1). To obtain an asporogenic phenotype, the gene encoding stage II sporulation protein AC, *spoIIAC*, was targeted for gene disruption. *spoIIAC* is a component of the SpoIIA-SpoIIQ type III secretion system residing in the forespore membrane, required for sigma factor SigG activation and, subsequently, the transcription of sporulation genes (32). Initial sequencing revealed that the region around the *spoIIAC* locus in B. subtilis KO7 was identical to the one annotated and deposited in the NCBI database for B. subtilis 168. We thus designed a construct to interrupt the *spoIIAC* gene, which would lead to a frameshift in the reading frame and cause an early termination of translation. The cassette was successfully cloned into pCC9X to give the final editing plasmid, pCC9X:sgRNA(*spoIIAC*) (Fig. 1B). After the transformation of pCC9X:sgRNA(*spoIIAC*) into KO7 cells, more than 100 CFU were obtained. Forty transformant colonies were screened by PCR, and >97% of them were shown to be likely mutated (see Fig. S1 in the supplemental material). The plasmid-cured KO7S2 strain showed a reverted sensitivity to kanamycin, as expected, and sequencing of the genomic region confirmed the correct mutation in *spoIIAC*. To further investigate its phenotype, single colonies of wild-type KO7 and the newly constructed KO7S2 strains were successfully grown in minimal medium to induce the formation of spores and subjected to heat shock in order to kill vegetative cells. In this study, KO7S2 was shown to lose heat resistance compared to wild-type KO7 (Fig. 2). After incubation on recovery medium, normal growth was observed for all dilutions of the untreated fraction for both the wild type and the mutant strain (Fig. 2). After heat treatment, colonies likely developing from the germination of spores were observed only for the wild-type strain, while no survivors were observed with the mutated strain (Fig. 2). These results confirm that the disruption of the *spoIIAC* gene led to an asporogenic phenotype (Fig. S2). Moreover, the results demonstrate the utility of the CRISPR/Cas9 editing plasmid for targeted gene engineering in *Bacillus*.

**TABLE 1:** Strains and plasmids used in this study

Strain or plasmid	Description	Source or reference
Strains		
E. coli MC1061	F^–^ λ^–^ Δ(*ara-leu*)*7697* [*araD139*]_B/r_ Δ(*codB-lacI*)*3 galK16 galE15* e14^–^ *mcrA0 relA1 rpsL150*(Str^r^) *spoT1 mcrB1 hsdR2*(r^–^ m^+^)	Laboratory strain
E. coli DB3.1	*gyrA462 endA1* Δ(sr1-*recA*) *mcrB mrr hsdS20 glnV44* (=*supE44*) *ara14 galK2 lacY1 proA2 rpsL20 xyl-5 leuB6 mtl-1*	Laboratory strain
B. subtilis KO7	PY79 Δ*nprE* Δ*aprE* Δ*epr* Δ*mpr* Δ*nprB* Δ*vpr* Δ*bpr*	D. R. Zeigler, *Bacillus* Genetic Stock Center (accession no. 1A1133)
B. subtilis KO7S2	Sporulation-free strain of KO7; Δ*spoIIAC*	This study

Plasmids		
pJOE8999	Kan^r^ *cas9* sgRNA *rep* pE194^ts^	17
pCC9X	pJOE8999 with an FX cassette replacing sgRNA	This study
pU57:sgRNA(*spoIIAC*)	SapI-free pUC57^*a*^ with a synthetic fragment containing the P_43_ promoter, an sgRNA, and homology arms toward *spoIIAC*	This study
pCC9X:sgRNA(*spoIIAC*)	pCC9X with sgRNA targeting *spoIIAC*	This study
pCC9X:sgRNA(*amy*E)	pCC9X with sgRNA targeting *amyE*	This study
pUC57:P*_xyl_*_A_-FX	SapI-free pUC57-kan^*a*^ with a synthetic BamHI-SalI fragment containing the *xylA* promoter and FX cassette	This study
pDG1662	Amp^r^ Spc^r^ Cm^r^ *amyE*	46
pDG1662Δ*spc*	PDG1662 without the Spc^r^ gene	This study
pUC57:*aprE*	SapI-free pUC57^*a*^ with E. coli codon-optimized subtilisin gene Bli01109 (*aprE*)	31
pAHX	pDG1662Δ*spc*^R^ with an FX cassette replacing Cm^r^	This study
pAHX:*aprE*	pAHX with E. coli codon-optimized subtilisin gene Bli01109 (*aprE*) replacing the FX cassette	This study

a See reference 30.

### A versatile double-plasmid system for CRISPR/Cas9-mediated targeted integration into the *Bacillus amyE* locus.

**FIG 3:**
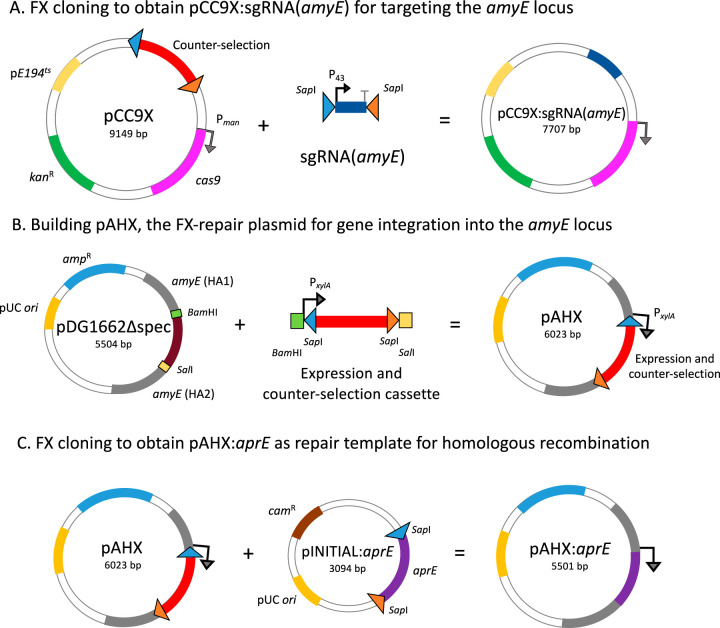
Efficient FX cloning for CRISPR-based editing in the *amyE* locus of B. subtilis. (A) An sgRNA is FX cloned into pCC9X to induce Cas9 cleavage in the *amyE* locus. (B) Building the repair plasmid pAHX by replacing the chloramphenicol resistance (Cam^r^) gene in pDG1662Δ*spc* with a BamHI/SalI-flanked fragment containing a xylose-inducible promoter (P*_xylA_*) and a *ccdB*-based counterselection cassette (red). The counterselection region is flanked with opposite outward-oriented SapI sites (triangles) to make it compatible with FX cloning. The homology arms (HA1 and HA2) (gray) in pAHX ensure homologous recombination of genes into the *amyE* locus of B. subtilis. (C) The repair plasmid pAHX serves as the destination for FX cloning of a gene of interest (here, *aprE* encoding subtilisin) from a delivery plasmid, pINITIAL. Gene lengths are not drawn to scale.

To establish a versatile engineering procedure for larger gene insertions, we designed a double-plasmid strategy to allow insertion into a fixed genomic position while avoiding large plasmid sizes. Such a system would complement our previous vector developments for high-throughput cloning and expression in E. coli (30) and *Bacillus* (31) and allow a fast transition to the stable integration of genes for production by fermentation. To allow the rapid and convenient screening of successful editing events, we selected the native *amyE* locus, which encodes a nonessential alpha-amylase, as the fixed ectopic integration site. Integration at the *amyE* locus will cause a disruption of the host’s ability to degrade starch, and clones with successful edits will manifest themselves on starch-supplemented agar plates as colonies lacking halos, in contrast to halo-forming wild-type amylolytic clones. The first plasmid of the double-plasmid system harbors the elements for CRISPR/Cas9-directed cleavage of the *amyE* locus. A P_43_ promoter-driven sgRNA, which directs a double-strand break in the *amyE* locus, was successfully assembled by PCR and subcloned into pCC9X, giving pCC9X:sgRNA(*amyE*) (Fig. 3A and Table 1). The second plasmid is a repair plasmid based on a reduced version of pDG1662 (Fig. S3). An inducible P*_xylA_* promoter, chosen for its tight regulation (33), followed by the FX-compatible *ccdB*-based counterselection cassette, was successfully cloned between the *amyE* homology arms in the pDG1662Δ*spc* backbone, resulting in the FX-compatible repair plasmid pAHX (Fig. 3B and Table 1). The plasmid harbors a customized gene expression cassette flanked by homology arms that will direct its integration into the *amyE* locus by homologous recombination and facilitate expression from the d-xylose-inducible promoter.

### Integration of a subtilisin-encoding gene into the bacterial chromosome using the double-plasmid system.

To validate the double-plasmid system, the Bacillus licheniformis *aprE* gene, encoding an extracellular subtilisin, was integrated into the chromosome of the newly constructed asporogenic B. subtilis KO7S2 strain. The resulting strain serves as a surrogate host for the expression of a protease from a chromosomal locus. Since the *aprE* gene of the KO7 strain had been deleted, along with other protease-encoding genes (Table 1), it was anticipated that the reintroduction of a homologous *aprE* gene would be tolerated. Moreover, since the recombinant subtilisin encoded by *aprE* is extracellular, downstream processing (purification) is simplified. Finally, the low protease background activity from the host strain was expected to minimize interference in measurements of proteolytic activity from the recombinant protease.

Previously, the *aprE* gene was constructed in the pINITIAL entry vector, known to produce a functional enzyme from plasmid-based expression in both E. coli (30) and *Bacillus* (31). The subtilisin-encoding gene cassette (*aprE*) was readily subcloned from pINITIAL:*aprE* into pAHX using FX cloning (Fig. 3C and Table 1). The resulting plasmid, pAHX:*aprE*, was cotransformed with pCC9X:sgRNA(*amyE*) into B. subtilis KO7S2, leading to a Cas9-promoted double-strand break of the *amyE* locus and integration of the *aprE* gene expression cassette by homologous recombination. A total of 48 colonies with diameters of >3 mm and 30 with diameters of <3 mm were obtained after overnight incubation. Twenty-five colonies of each colony size were transferred to a starch plate to screen for the development of starch-degrading clearing zones (Fig. S4). None of the larger clones produced halos, indicating the loss of amylolytic activity and successful integration (Table 2), whereas 3 of the smaller colonies produced a clearing zone.

**TABLE 2:** Results obtained with the double-plasmid system for the integration of *aprE* into the B. subtilis KO7S2 chromosome

Expt	Vector(s) used for transformation	No. of colonies of >3 mm	No. of colonies with negative amylase phenotype on starch plates/total no. of colonies	No. of confirmed integrated mutants by PCR screen/total no. of mutants (%)
Control	pCC9X:sgRNA(*amyE*)	0		
*aprE*	pCC9X:sgRNA(*amyE*) + pAHX:*aprE*	48	25/25	8/8 (100)

Chromosomal gene integration was confirmed by PCR for eight randomly selected colonies. PCR screening of 8 of the 22 small colonies that seemed to have lost amylolytic activity did not generate a product, indicating a disruption of the *amyE* gene. A control experiment without the repair plasmid also resulted in small colonies after overnight incubation, all with diameters of <3 mm. Thirty-two of these colonies were streaked on starch-supplemented agar plates. A clearing zone was observed after 24 h for 5 colonies, indicating retained amylolytic activity, as expected. Halos were not observed for the other 27 colonies, suggesting that the *amyE* gene was disrupted. PCR screening of 7 of these halo-negative colonies showed 2 *amyE* gene amplicons of shorter lengths, whereas 5 did not result in a product (Fig. S4). A control with pAHX only was not included since its lack of functional selection markers in *Bacillus* would not provide any readable results. Four mutant clones were subjected to heat treatment for plasmid curing, and successful plasmid loss was verified by reverted sensitivity to kanamycin. Moreover, correct integration was confirmed by sequencing of the genomic region. The new strain was named KO7S2 *amyE*::*aprE* (*aprE* inserted in the *amyE* locus).

### Gene engineering had no impact on growth rates obtained through microbial fermentation.

**FIG 4:**
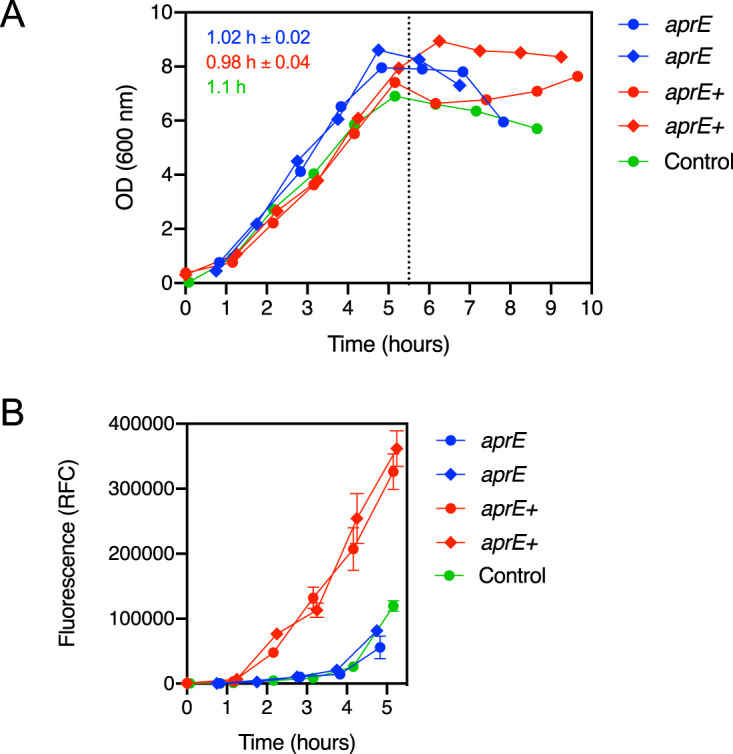
Batch fermentation with *Bacillus* KO7S2 *amyE*::*aprE*. (A) OD_600_ of fermentations of the KO7S2 strain (control) and the *aprE*-containing KO72S *amyE*::*aprE* strain (two uninduced replicates, *aprE*, and two induced replicates, *aprE+*). The doubling times in hours (*T_d_*) are inserted into the chart with the corresponding color codes. No significant differences in cell density were observed over time between strains, even after the induction of recombinant subtilisin expression. The dotted vertical line shows the transition from exponential phase to stationary and/or death phase. (B) Proteolytic activity measured as relative fluorescence counts (RFC) from supernatants of the fermentation cultures in panel A during exponential phase (<5.5 h). Color codes are the same as the ones for panel A. Although uninduced and *aprE*-negative strain controls resulted in substantial proteolytic activity in the extracellular medium toward the end of the exponential phase, the response was delayed more than 2 h compared to the induced strain, altogether suggesting the induced expression of AprE from KO72S *amyE*::*aprE*.

To assess the effects of the chromosomal integration of *aprE* on microbial growth and to monitor the production of a functional subtilisin, fermentation experiments were carried out in bench-scale reactors. The growths of *Bacillus* KO7S2 *amyE*::*aprE* and KO7S2, the latter serving as an *aprE*-negative control in this experiment, were compared to that of d-xylose-induced *Bacillus* KO7S2 *amyE*::*aprE*. Overall, the growth curves obtained in the reactors for the different strains and conditions were comparable with regard to the duration of the lag phase, which was minimal, and all of them reached the stationary or death phase within 5 to 6 h (Fig. 4A). The calculated doubling times (*T_d_*s) for KO7S2 and both KO7S2 *amyE*::*aprE* cultures were also found to be comparable (Fig. 4A). The proteolytic activity was monitored in the media of all batch fermentations during lag and exponential phases (Fig. 4B). As expected, based on the presence of the endogenous WprA protease, substantial proteolytic activity was observed in the control parent KO7S2 strain after 4 h as the culture entered the stationary phase. A similar pattern was observed for the uninduced KO7S2 *amyE*::*aprE* strain. On the contrary, proteolytic activity was observed with the induced KO7S2 *amyE*::*aprE* strain starting about 2.5 h earlier, strongly suggesting induced expression of AprE. Moreover, the proteolytic activity continued to increase exponentially, congruent with the growth curve (Fig. 4B). Altogether, these results suggest that KO7S2 *amyE*::*aprE* produces a functional AprE subtilisin. Proteolytic activity for the uninduced and parent KO7S2 strains was observed only after about 4.5 h (Fig. 4B) at levels comparable to that of the induced KO7S2 *amyE*::*aprE* strain at 2 h.

## DISCUSSION

CRISPR/Cas9-based systems for targeted genome engineering in *Bacillus* have previously been shown to work more efficiently than other markerless methods, mostly due to the strong selection toward correctly repaired mutant clones (15, 17). However, the requirement of specific spacer sequences for each engineering step is a disadvantage since it delays the construction of the final editing vectors (16, 17). Taking advantage of the flexibility and efficiency of FX cloning and equivalent assembly cloning methods based on type IIS restriction enzymes (28), we adapted a plasmid suite for the easy and efficient construction of CRISPR/Cas9-mediated genome-editing tools. The systems allow fast transitions from HTP protein expression to production by fermentation of B. subtilis. The customized tools were validated by engineering a *Bacillus* strain suitable for the production of recombinant enzymes through microbial fermentation. The adapted vector tools will allow versatile editing at any chosen genomic position (single-plasmid strategy) or at a fixed genomic *amyE* locus (double-plasmid strategy) with minimal seams. The FX-compatible shuttle vector pCC9X was as efficient as its parent pJOE8999 vector (17) (Table 2), but obtaining the final editing vector can now be accomplished in one single and efficient cloning step (Fig. 1 and 3), thus significantly shortening and simplifying construct engineering (15). Moreover, an editing cassette can also be assembled in a linear order and be readily cloned into pCC9X to obtain the final editing plasmid.

The double-plasmid strategy (Fig. 3) led to the successful insertion of *aprE* into the *amyE* locus with minimal seams. The appearance of background colonies in the control experiments that do not include the repair plasmid is not fully understood. The repair of the Cas9/sgDNA-directed DNA cleavage of *amyE* disagrees with the assumption that B. subtilis lacks a nonhomologous end-joining (NHEJ) system (15). Cas9-resistant colonies may be explained by spontaneous mutants with a defective Cas9 protein (15) or kanamycin-resistant cells. The GC content of the sgRNA, particularly 6 bp proximal to the protospacer-adjacent motif (PAM), has been shown to be important for efficient cleavage (34, 35). Although better-GC-scored protospacer sequences were predicted in the central part of the *amyE* locus, the position relative to the 5′ end was prioritized to minimize the risk of production of a partial AmyE. Despite the background, the efficiency of the integration system is very high and comparable to those of other similar CRISPR/Cas9 methods previously described (15–17), mainly due to the positive selection of mutants.

To validate the FX-compatible editing plasmid pCCX9 we chose to knock out a key gene, *spoIIAC*, involved in sporulation (Fig. 2). The formation of highly resistant spores is an undesirable property that may hamper the application of *Bacillus* as a microbial cell factory to produce enzymes (36). It was previously reported that mutations in some sporulation genes, such as *spo0A*, result in higher intracellular fluxes of metabolites and increased biomass yields (37). The results of this study show that the loss of sporulation in the *spoIIAC*-null mutant does not hamper exponential growth (Fig. 4; see also Fig. S5 in the supplemental material), in line with previous reports (37).

We chose the subtilisin-encoding *aprE* gene for validation of the double-plasmid system for editing in a fixed *amyE* position, based on the assumptions that loss-of-function mutations of *amyE* would provide an easy screening method (38) and that reintroducing the AprE subtilisin would be a well-tolerable target. The rapid increase in proteolytic activity from xylose-induced *Bacillus* KO7S2 *amyE*::*aprE* follows 5 h of exponential-phase growth (Fig. 4) and suggests that the chromosomally integrated AprE subtilisin is functional, as only subtle activity is detected in the uninduced control and the parent KO7S2 strain during the same interval (Fig. 4B). The delayed increase in proteolytic activity in both controls, starting in the midst of the stationary phase, is likely due to cell lysis with the concomitant release of WprA protease (9). This is further supported by the heavy foaming observed during the stationary phase (data not shown), which is known to be enhanced by cell lysis (39).

**FIG 5:**
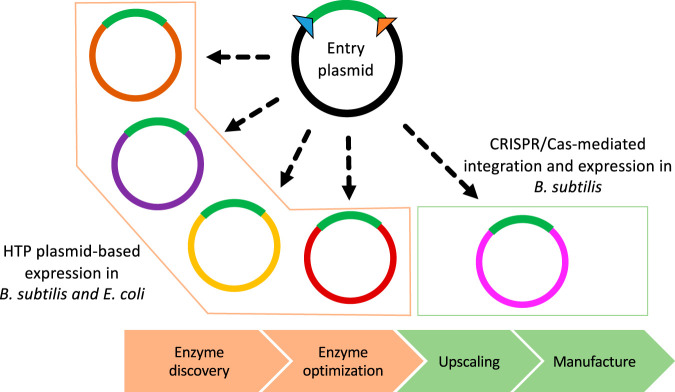
Overview of the compatible FX cloning platform for quick transitions from high-throughput cloning and the expression of new enzymes in E. coli and B. subtilis (enzyme discovery and optimization phases) to stable CRISPR/Cas-mediated integration of genes for the production of enzymes through B. subtilis fermentation (upscaling and manufacture).

Overall, the customized plasmid tools described here allow a convenient cloning procedure for marker-free engineering with minimal seams. The system is compatible with previous high-throughput cloning procedures and allows one-step subcloning to move from plasmid-based expression (30, 31) to stable chromosome integration and expression in a production strain in less than a week (Fig. 5). As demonstrated by the example with subtilisin, the customized plasmid tools facilitate a quick transition from the discovery and characterization phases to upscaled enzyme production and manufacturing by microbial fermentation (Fig. 5). A native integration site, such as the *amyE* locus, does not require any preparative modifications of the host prior to editing, as opposed to artificially constructed integration sites (12). Moreover, the *amyE* locus is conserved among multiple *Bacillus* strains, making the editing vectors universal to multiple strains (16). These are all highlighted as attractive properties for editing tools (40). Based on the demonstration that other gain-of-function or loss-of-function target genes (38, 41) are useful for editing and rapid mutant screening in *Bacillus*, we are confident that the adapted CRISPR/Cas9 tools are versatile and applicable for a variety of engineering events.

## MATERIALS AND METHODS

### Strains and media.

The bacterial strains and plasmids used in this study are listed in Table 1. E. coli DB3.1 and MC1061 were used to propagate recombinant plasmids. All B. subtilis strains were derived from B. subtilis PY79 by using KO7, a strain with markerless clean deletions of seven extracellular proteases (Zeigler, unpublished), which is available from the *Bacillus* Genetic Stock Center (BGSC) (accession no. 1A1133; Ohio State University). Unless otherwise indicated, all reagents were purchased from Sigma-Aldrich. Bacterial cells were grown in lysogeny broth (LB) medium (1% [wt/vol] tryptone, 0.5% [wt/vol] yeast extract, and 0.5% [wt/vol] NaCl) or LB agar medium (supplemented with 1.5% [wt/vol] agar). Fermentation medium was composed of 5 g/liter (wt/vol) yeast extract (Acros Organics, NJ, USA), 10 g/liter (wt/vol) soy peptone (Sigma-Aldrich, Germany), and 10 g/liter (wt/vol) NaCl (VWR Chemicals, Belgium). The following antibiotics were added to the media when required: ampicillin (100 μg/ml), chloramphenicol (15 μg/ml), and kanamycin (10 or 50 μg/ml).

### Construction of an FX-cloning-ready plasmid for CRISPR/Cas9-mediated genome editing in B. subtilis.

DNA oligonucleotides were purchased from Merck (Table 3). Synthetic genes were purchased from GenScript. The reagents for Phusion polymerase PCR, restriction enzymes, and DNA phosphorylation and ligation were purchased from New England BioLabs (NEB). PCR and plasmid purifications were done with a PCR cleanup kit and a plasmid miniprep kit (Macherey-Nagel, Germany). Plasmid pJOE8999 (17) was purchased from the BGSC. The native SapI site in pJOE8999 was removed by site-directed mutagenesis using the overlapping primer set MUT7977-F and MUT7968-R, as it was in conflict with the FX cloning strategy. PCR amplification was done with Phusion DNA polymerase according to the manufacturer’s recommendations. Correct amplification products of the expected size were confirmed by agarose gel electrophoresis. Parental template plasmid DNA was eliminated by digestion with DpnI at 37°C for 1 h, and chemically competent E. coli MC1061 cells were transformed and plated onto LB agar supplemented with 50 μg/ml kanamycin for selection. The correct SapI-free mutant plasmid was confirmed by sequencing with primer SQ7906-F. The FX counterselection cassette was amplified from the previously described vector p12 (30) using the primer set MP-F1 and MP-R1. The megaprimer amplicon of approximately 1.8 kb was purified and cloned into the previously obtained SapI-free backbone of pJOE8999 by exponential megapriming PCR (EMP-PCR) (42) in combination with primer EX-R2. Phosphorylation, circularization, and digestion of the parental plasmid were done as described in the same work. The final plasmid construct, pCC9X (plasmid CRISPR/Cas9 FX), was then transformed into chemically competent E. coli DB3.1 and plated onto LB agar supplemented with 50 μg/ml kanamycin for selection. Sequencing using primers 251SQ_FXcam-F and 91SQ_FXcam-R (Table 3) was used to verify the correct assembly of pCC9X.

**TABLE 3:** Primers used in this study

Primer	Sequence^*a*^	Purpose
BsamyE-F2	5′-CCAAACTGGACACATGGAAAC	Sequencing primer
BsamyE-R3	5′-GAAAAGAGGCGTACTGCCTG	Sequencing primer
91_SQccdB-R	5′-GAAAATGACATCAAAAACGCCATTAACC	Sequencing primer
MP-F1	5′-AAAAGGCCCAGTCTTTCGACTGGGCAGTAGAAGAGCGAGCTGCAGACTGG	Megaprimer F
MP-R1	5′-GAATTCGTAATCATGGTCATAGCTGTGCAGAAGAGCTGAACTAGTG	Megaprimer R
EX-R2	5′-TAATACGACTCACTATAGGG	EMP-PCR R-primer
251SQ_FXcam-F	5′-CATTTTACGTTTCTCGTTCAGCTTTTTTG	Sequencing primer
91SQ_FXcam-R	5′-GAAAATGACATCAAAAACGCCATTAACC	Sequencing primer
MUT7977-F	5′-AGAAGAACTGTTTGAATATGC	Mutagenesis primer
MUT7968-R	5′-AAACAGTTCTTCTACGATAAGG	Mutagenesis primer
SQ7906-F	5′-GAGAAAGTGTATCAAACTGC	Sequencing/screening primer
Bs2444359-F	5′-TGCTTCATTTATAGCTCAGC	Sequencing/screening primer
Bs443156-R	5′-AAGACCATAAATTACCACGC	Sequencing primer
Screen-R	5′-TGTTTAAAAACCGCCTCGAG	Screening primer
p43_SapI-F	5′-GCTCTTCTAGTATTTTACATTTTTAGAAATGGGCG	Assembly PCR
p43_amyE-R	5′-TCGATCAGACCAGTTTTTAATTATATTTTACATAATCGCGCG	Assembly PCR
term_amyE-F	5′-TTAAAAACTGGTCTGATCGAGTTTTAGAGCTAGAAATAG	Assembly PCR
term_SapI-R	5′-GCTCTTCATGCGCCTAAGCTTTAGATAAAAAACGCC	Assembly PCR
T7p-R	5′-CCTATAGTGAGTCGTATTA	Sequencing
M13-F	5′-TGTAAAACGACGGCCAGT	Sequencing

a Restriction sites are underlined.

### CRISPR/Cas9-mediated disruption of *spoIIAC*.

To disrupt the *spoIIAC* gene in B. subtilis KO7, a 1,253-bp-long editing cassette, flanked by two unique SapI sites for directed FX cloning, was designed and synthesized (28). The cassette contains a strong constitutive P_43_ promoter for the translation of an sgRNA in B. subtilis and a repair template with two 500-bp-long homology arms against the B. subtilis genome, flanking the insertion point in the *spoIIAC* locus. The P_43_ promoter, scaffold DNA, and terminator for sgRNA were taken from pJOE8999. The *spoIIAC*-specific 20-nucleotide (nt) protospacer 5′-TTGTTTGGTCTGTCGTACAG-3′ was designed to match the region upstream of the protospacer-adjacent motif (PAM) CGG located at positions 151 to 153 within the *spoIIAC* gene, as annotated for B. subtilis 168. This PAM was selected based on its position relative to the 5′ gene start site to ensure the early disruption of translation, the lack of off-target effects against the B. subtilis genome by BLAST (43), and the percent GC score of the DNA sequence (overall 45% GC and 50% GC contents within the 6 bp proximal to the 3′ end, and a G just before the PAM), according to previously recommended guidelines (34, 35). Homologous recombination introduces an 8-bp insertion that disrupts the reading frame, resulting in the early termination of translation and a truncated, dysfunctional SpoIIAC. The synthetic editing cassette was delivered in a customized SapI-free pUC plasmid carrying a selection marker against ampicillin (GenScript). The editing cassette was inserted into pCC9X by FX cloning as described previously (28, 30). The final editing plasmid, pCC9X:sgRNA(*spoIIAC*), was propagated in E. coli MC1061 cells on LB agar with 50 μg/ml kanamycin for selection. Colony PCR was conducted to screen for the correct construct using primers M13-F and T7p-R. Genome editing was carried out using naturally competent B. subtilis KO7 cells (31) with 500 ng pCC9X:sgRNA(*spoIIAC*). Transformants were spread on LB agar plates supplemented with 10 μg/ml kanamycin and 0.2% (wt/vol) d-mannose for inducing the expression of the Cas9 protein and further incubated overnight at 30°C as described previously (17). Positive transformants were transferred to a new antibiotic-free LB agar plate and incubated overnight at 50°C for plasmid curation by inhibiting the temperature-sensitive replicon. Kanamycin-sensitive clones were screened, using colony multiplex PCR with primers Bs2444359-F, Bs2443156-R, and Screen-R (Table 3), for the presence of a disrupted *spoIIAC* gene. Primers Bs244359-F and Bs244356-R match against the region flanking the *spoIIAC* locus, while the internal primer Screen-R matches against the 8-bp insertion that disrupts *spoIIAC* in the knockout mutants. Amplification with the three primers produces a double band of 1.3 kb and 0.5 kb in knockout mutants but a single band of 1.3 kb in wild-type colonies. A positive knockout mutant colony was streaked out on LB agar and further incubated overnight at 50°C. Individual colonies were tested for kanamycin sensitivity, and genomic DNA was extracted for sequencing of the *spoIIAC* locus. The new asporogenic strain was named B. subtilis KO7S2 and was used in further work.

### Validating the asporogenic KO7S2 strain.

The new KO7S2 strain was tested for its ability to produce resistant spores under stress conditions and compared against the parent strain KO7S. Both strains were inoculated in sporulation minimal medium (0.5% [wt/vol] tryptone, 0.25% [wt/vol] yeast extract) and incubated at 40°C for 48 h to induce sporulation. Serial dilutions were subsequently prepared, and each dilution was split into two fractions: one of them was subjected to heat treatment at 75°C for 20 min to kill vegetative cells, while the other was kept as a control. Both fractions (heat treated and nontreated) were plated onto LB agar recovery medium and further incubated overnight at 37°C. The sporulation efficiency for each dilution was calculated as the number of CFU from germinated spores in the heat-treated fraction divided by the total number of CFU in the nontreated fraction. Additionally, the growth performances of both KO7 and KO7S2 were compared using batch cultures. Individual colonies from LB agar plates were precultured overnight in LB medium at 30°C. Culture volumes of 200 ml of LB medium in 1-liter Erlenmeyer flasks were inoculated with 2 ml of each preculture and further incubated at 37°C under constant agitation at 250 rpm. Samples were taken at regular intervals, and the optical density at 600 nm (OD_600_) was determined in duplicate using a Sense microplate reader (Hidex, Finland). OD_600_ values were plotted, a linear regression model was adjusted in the exponential growth phase, and the doubling time was inferred.

### Construction of plasmids for *amyE*-directed chromosomal integrations.

A P_43_ promoter-driven sgRNA targeting *amyE* was constructed by assembly PCR using the primer set p43_SapI-F and p43_amyE-R and the primer set term_amyE-F and term_SapI-R (Table 3). To allow a minimal 500-nt length of the upper and lower homology arms in the repair template plasmid, the location of the protospacer had to be restricted to the central 1,000-nt stretch in the *amyE* gene locus. Therefore, for the disruption of the *amyE* gene, a 20-nt protospacer sequence, 5′-TTAAAAACTGGTCTGATCGA-3′, upstream from the PAM 5′-TGG-3′ located at positions 523 to 525 within the *amyE* gene, was predicted using Web-based tools (44, 45). Few off-target effects were predicted for the genome of B. subtilis 168. The PCR product was purified, FX cloned into pCC9X, transformed into MC1061 cells, and plated onto LB agar with 50 μg/ml kanamycin. The correct assembly of pCC9X:sgRNA(*amyE*) was verified by sequencing using the primer pair T7p-R and M13-F (Table 3). The plasmid pDG1662 (BGSC) was chosen as a scaffold for the repair plasmid to obtain marker-free ectopic integration into *Bacillus* (46). The native *xylA* promoter (P*_xylA_*) from B. subtilis 168 (33) was designed as a genetic fusion to the FX counterselection cassette (28) flanked by BamHI and SalI restriction sites. The P*_xylA_*-FX cassette was synthesized in a customized SapI-free pUC57 plasmid (GenScript). In order to reduce the overall plasmid size and secure a marker-free recombination event, the irrelevant spectinomycin gene was removed from pDG1662 by digestion with SacI and XhoI. The resulting plasmid, pDG1662Δ*spc*, and the synthetic P*_xylA_*-FX cassette were digested with BamHI and SalI, and the two relevant fragments were gel purified and ligated. The resulting plasmid, pAHX (plasmid *amyE* homology FX), was transformed in chemically competent *ccdB*-resistant E. coli DB3.1 cells and selected on LB agar with 100 μg/ml ampicillin. Correct cloning was identified by colony PCR with primers BsamyE-F2 and BsamyE-R3 (Table 3). Primers BsamyE-F2, BsamyE-R3, and 91_SQccdB-R (Table 3) were used to verify the correct assembly of pAHX by sequencing. As this plasmid cannot replicate in *Bacillus* (46), it thus serves as a repair template for the homologous recombination of the gene of interest that it carries into the *amyE* locus.

### CRISPR/Cas9-mediated integration of *aprE* into the *amyE* locus.

A codon-optimized *aprE* gene encoding full-length subtilisin (including the native leader sequence) from B. licheniformis (31) was FX cloned into pAHX (28) to obtain pAHX:*aprE*. After confirming the correct construct by colony PCR with primers BsamyE-F2 and BsamyE-R3 (Table 3), a clone was propagated for plasmid DNA isolation. Genome editing was carried out using naturally competent B. subtilis KO7S2 cells as described previously (31), by cotransformation with 500 ng of pCC9X:sgRNA(*amyE*) and 500 ng of the pAHX:*aprE* repair plasmid. A control experiment omitting the repair plasmid was included. Transformants were selected on LB agar plates supplemented with 10 μg/ml kanamycin and 0.2% (wt/vol) d-mannose and incubated for 16 h at 37°C. Colonies with a diameter of >3 mm were transferred to a new LB agar plate supplemented with 10 μg/ml kanamycin and 1% (wt/vol) soluble potato starch. After incubation at 37°C for 24 h, positive *amyE* knockout mutants lacked a clear halo around the colony due to the loss of the amylolytic phenotype. Successful recombination was checked by colony PCR using primer set BsamyE-F2 and BsamyE-R3 (Table 3). Two consecutive incubations at 50°C were done to cure the strain of the plasmid, and positive mutant clones were tested for the expected restored sensitivity to kanamycin and ampicillin. The correct recombination of *aprE* into the *amyE* locus was confirmed by sequencing with primers BsamyE-F2 and BsamyE-R3.

### Batch fermentation and recombinant expression of subtilisin.

Batch fermentation experiments were performed using two different B. subtilis strains, namely, KO7S2 Δ*spoIIAC* (*spoIIAC*-null mutation that confers an asporogenic phenotype) and KO7S2 *amyE*::*aprE* (asporogenic strain with a subtilisin gene inserted into the chromosomal *amyE* locus). The pH was adjusted to 6.8. Glanapon 2000 (Bussetti, Austria) was used as antifoam in all experiments. Batch fermentations were carried out in a 16-liter turbine-stirred bioreactor (NLF-16 Bioengineering AG, Wald, Switzerland). Up to 8.0 kg of sterilized medium was used in each fermentation experiment. For induction, 250 ml of a d-xylose solution was added to the fermentor (final concentration of 0.5% [wt/vol]) after the cell density (OD_600_) reached 0.5 to 0.6. Batch fermentation was started by adding 500 g of an inoculum culture grown at 37°C at 180 rpm for 7 h in LB medium. During cultivation, the temperature in the reactor was kept at 37°C by means of a water cooler (VC 3000, Variocool; Lauda, Germany) and steam. The pH of the culture was monitored through an inbuilt pH probe and maintained at 6.8 by adding 2 M NaOH or 6 M H_3_PO_4_. The aeration rate was maintained at 1.0 volume of air per volume of liquid per min (VVM) via a mass flow controller by supplying sterile air. Dissolved oxygen tension was measured and controlled at a minimum of 30% air saturation by regulating the stirrer speed during the experiments. The exhaust gas of the fermentor was analyzed online for CO_2_ and O_2_ volume fractions by the inbuilt gas analyzer. Online data acquisition was carried out using BioSCADA laboratory software. The batch phase was considered finished and cultivation was stopped when a permanent drop in the off-gas CO_2_ concentration followed by a reduction of the OD_600_ were observed, approximately 6 h after inoculation. The batch experiments were performed in duplicates, and samples were taken aseptically.

### Batch fermentation monitoring.

Samples were collected every 30 min during batch experiments. Cell growth was monitored by measuring the cell density at an OD_600_ using a UV-visible spectrophotometer (Ultraspec 2100 pro; Amersham Biosciences, Uppsala, Sweden). The maximum growth rate for each strain was calculated based on the off-gas CO_2_ profile obtained during its batch cultivation. The doubling time was calculated from the growth rate. Subtilisin activity was analyzed by fluorescein isothiocyanate (FITC)-labeled casein degradation (Sigma-Aldrich) as described previously (30). Assay mixtures contained 5 μl of the cleared fermentation broth, 25 μl of 50 mM Tris-HCl (pH 8.5), and 20 μl of FITC-labeled casein. After incubation at 37°C for 1 h, the reactions were stopped by adding 150 μl 0.6 N trichloroacetic acid (TCA) to the mixture. Insoluble material was removed by centrifugation at 5,000 × *g* for 30 min at 4°C. The supernatant was used to measure fluorescence with excitation at 485 nm and emission at 520 nm using a Sense microplate reader (Hidex, Turku, Finland). Two technical replicates were run for each time point.

## Supplementary Material

Supplemental file 1
