# Supplementary material for: Fragment Exchange Plasmid Tools for CRISPR/Cas9-Mediated Gene Integration and Protease Production in Bacillus subtilis
Source: Appl Environ Microbiol. 2020 Dec 17;87(1):e02090-20. doi: 10.1128/AEM.02090-20 (PMC7755240; doi:10.1128/AEM.02090-20)
Supplement: Supplemental file 1 [file AEM.02090-20-s0001.pdf]

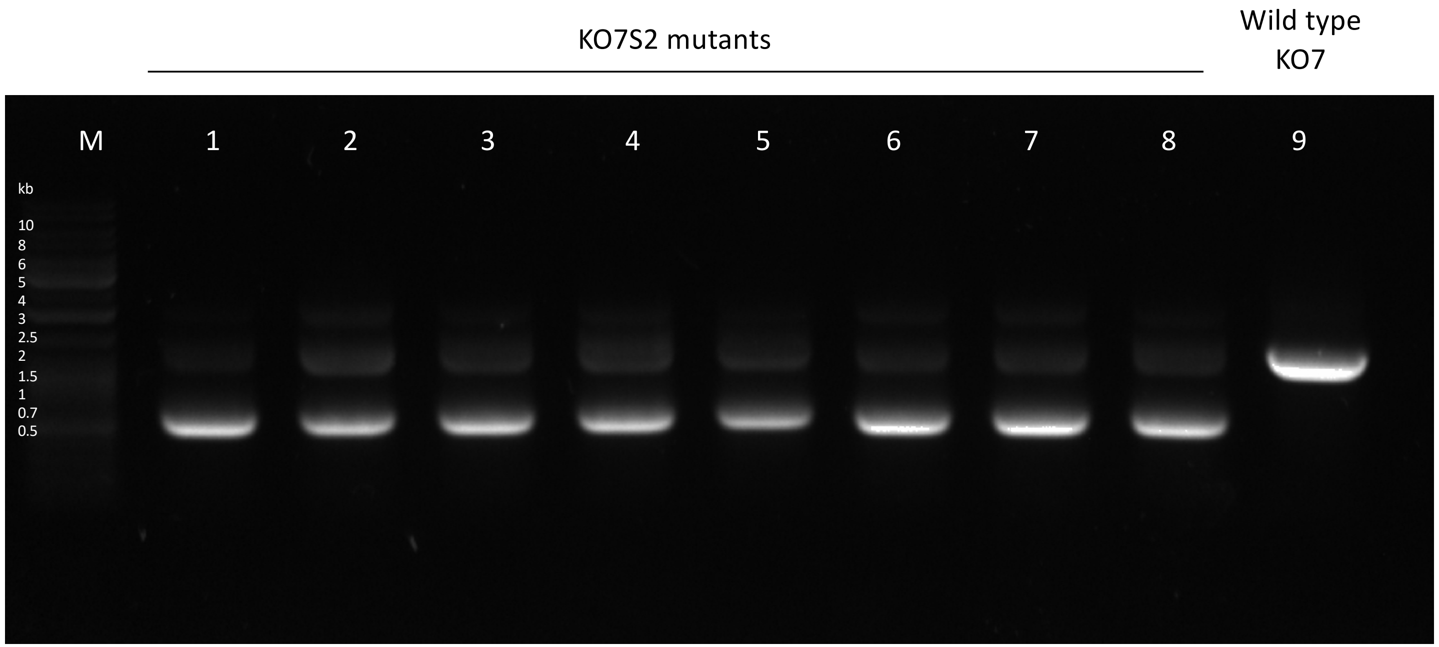

Figure S1. Colony PCR screen for *spoIIAC* knockout mutant. After successful recombination, an 8-bp insert was introduced into the *spoIIAC* gene locus, thus shifting the reading frame and producing an early termination of transcription. The correct mutant was identified with a multiplex PCR screen using two flanking primers and one targeting the insertion sequence. A positive knockout mutant will produce a double amplicon resulting from the amplification of Bs2444359-F with Bs443156-R (ca 1.2 kb) and Bs2444359-F with Screen-R (around 0.6 kb). The wild type strain will produce one single amplicon from Bs2444359-F and Bs443156-R (around 1.2 kb). This agarose gel picture shows the results of eight randomly picked colonies (lanes labelled 1-8) and a negative control with the wild type KO7 strain (lane 9). The lane labelled as M is the DNA size marker in kb.

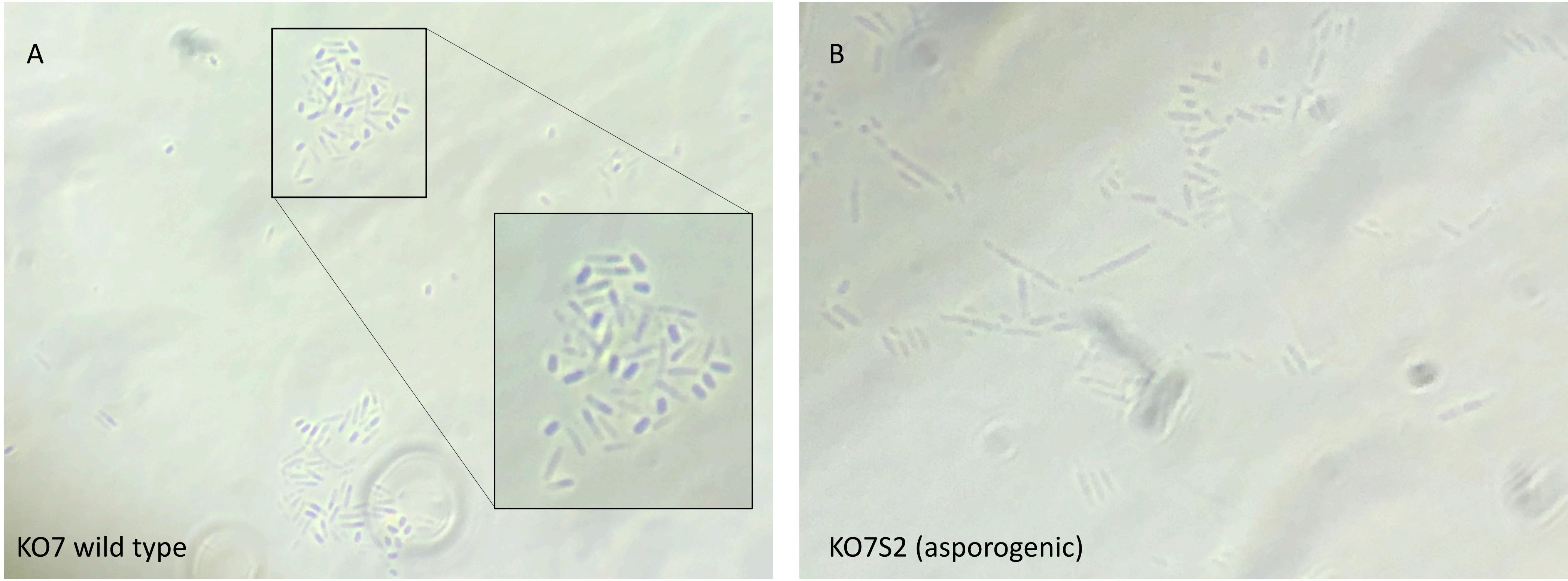

Figure S2. Phase contrast microscope images of the two *Bacillus* strains KO7 and KO7S2 after growth in sporulation minimal medium at 40 °C for 48 hours. (A) Endospores in the wild type *Bacillus* KO7 strain are recognized by their intracellular site of formation and increased refractility. (B) Endospores are absent in the asporogenic, *spoIIAC* deficient KO7S2 *Bacillus* strain.

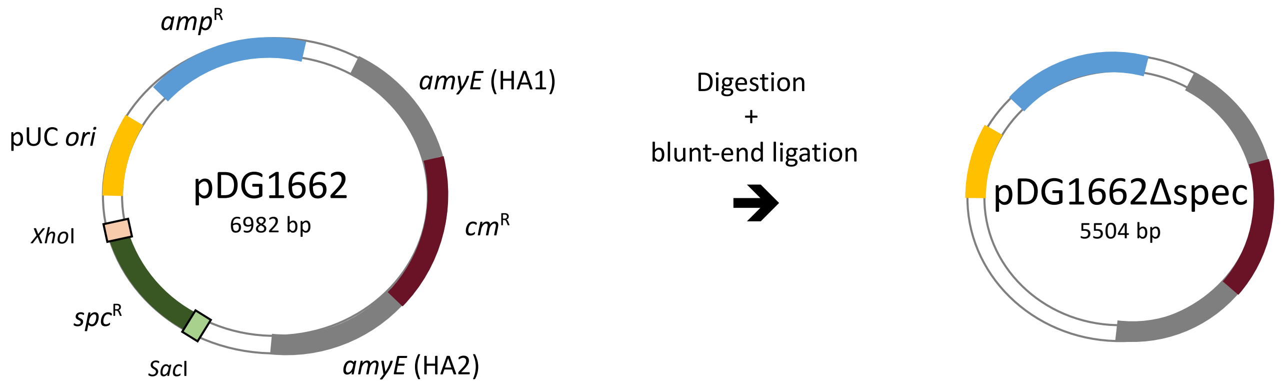

Figure S3. Constructing the repair plasmid. The overall size of pDG1662 is reduced by eliminating the spectinomycin resistance marker. The plasmid is subjected to double digestion with the one-cutter *Sac*I and *Xho*I. The backbone is gel-purified, end-repaired digested and blunt ligated to produce pDG1662Δ*spc*, which serves as backbone for developing the FX-adapted repair plasmid pAHX.

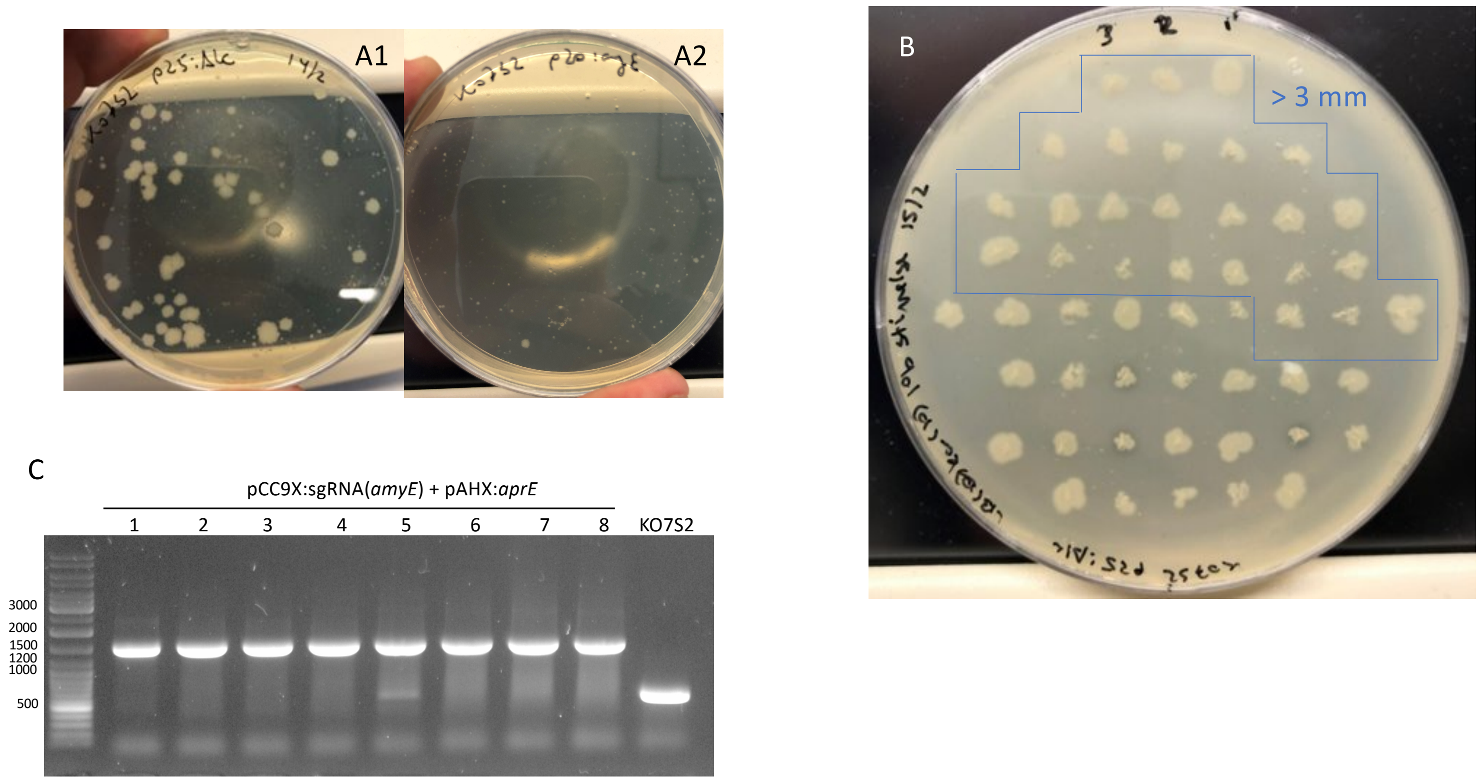

Figure S4. Insertion of *aprE* into the *amyE* locus using the double-plasmid system. (A) Transformants were selected on LB agar plates supplemented with 10 µg/mL kanamycin and 0.2 % (w/v) D-mannose and incubated for 16 h at 30 ° C. Co-transformation with pCC9X:sgRNA(*amyE*) and the repair pAHX:*aprE* plasmid, resulted in two easily distinguishable sized populations (A1) while the control experiment without repair template produces only small <3 mm colonies (A2). (B) All large >3 mm colonies (1 to 25) show loss of amylolytic phenotype on LB agar plates supplemented with 10 ug/mL kanamycin and 1 % (w/v) soluble potato starch. (C) Colony PCR screen with primers BsamyE-F2 and BsamyE-R3 show integration of the *aprE* gene cassette (ca. 1.4 kb amplicon) in 8 randomly picked colonies (lanes 1-8) while the negative wild-type strain KO7S2 renders an amplicon corresponding to the native *amyE* gene (ca. 0.5 kb). The clone in lane 5 is likely a mix population.

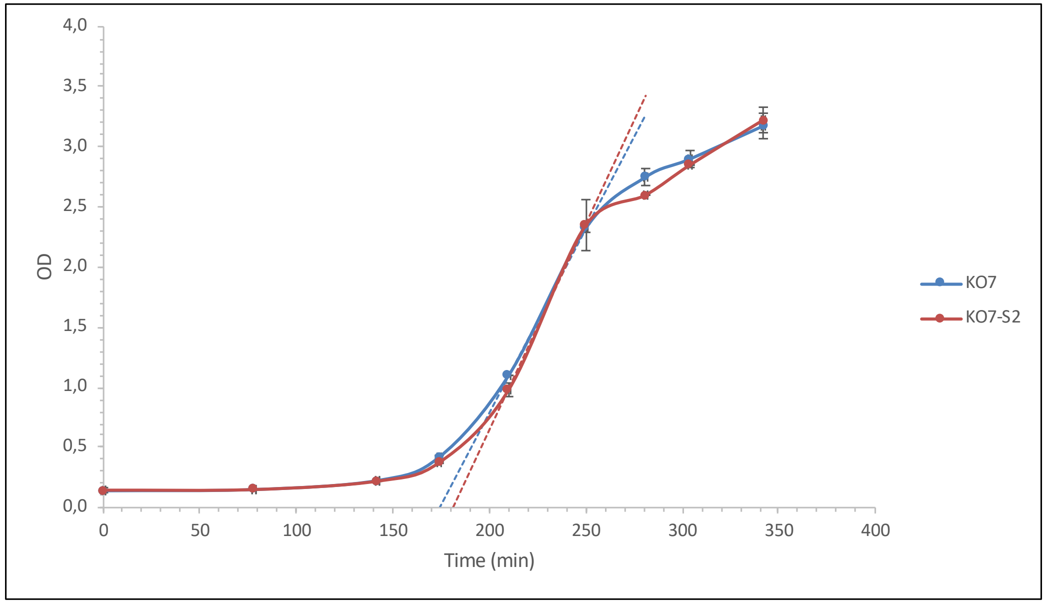

Calculating doubling time between 210 and 250 min by using the following regression lines

KO7
y1 = 0,0313x - 5,4625
y2 = 0,03x - 5,2

KO7S2
y1 = 0,039x - 7,25
y2 = 0,0295x - 5,175

Doubling times are then calculated to be 35.9 +/- 1.1 min for KO7 and 29.5 +/- 6.2 for KO7S2.

Figure S5. Batch culture growth performance of the asporogenic *spoIIAC* knockout mutant KO7S2, compared to KO7. A culture volume of 200 ml LB medium in a 1 L Erlenmeyer flask, was inoculated with 2 ml of each preculture and further incubated at 37 ° C under constant agitation at 250 rpm. Samples were taken at regular intervals and optical density at 600 nm (OD_{600}) determined in duplicate. OD_{600} values were plotted, a linear function was adjusted in the exponential growth phase and the doubling time was inferred. The experiment was carried out twice. The growth curves and doubling times for both strains were comparable, confirming that the null mutation has no deleterious effect in KO7S2.
